# Phase stabilization of cesium lead iodide perovskites for use in efficient optoelectronic devices

**DOI:** 10.1038/s41427-024-00540-0

**Published:** 2024-05-03

**Authors:** Handong Jin, Yu-Jia Zeng, Julian A. Steele, Maarten B. J. Roeffaers, Johan Hofkens, Elke Debroye

**Affiliations:** 1 Department of Chemistry, KU Leuven, Leuven, Belgium; 2 Key Laboratory of Optoelectronic Devices and Systems of Ministry of Education and Guangdong Province, College of Physics and Optoelectronic Engineering, Shenzhen University, Shenzhen, People’s Republic of China; 3 Australian Institute for Bioengineering and Nanotechnology and School of Mathematics and Physics, The University of Queensland, Brisbane, QLD Australia; 4 cMACS, Department of Microbial and Molecular Systems, KU Leuven, Leuven, Belgium

**Keywords:** Optical materials, Electronic devices

## Abstract

All-inorganic lead halide perovskites (LHPs) and their use in optoelectronic devices have been widely explored because they are more thermally stable than their hybrid organic‒inorganic counterparts. However, the active perovskite phases of some inorganic LHPs are metastable at room temperature due to the critical structural tolerance factor. For example, black phase CsPbI_3_ is easily transformed back to the nonperovskite yellow phase at ambient temperature. Much attention has been paid to improving the phase stabilities of inorganic LHPs, especially those with high solar cell efficiencies. Herein, we discussed the origin of phase stability for CsPbI_3_ and the strategies used to stabilize the cubic (α) phase. We also assessed the CsPbI_3_ black β/γ phases that are relatively stable at nearly room temperature. Furthermore, we determined the relationship between phase stabilization and defect passivation and reviewed the growing trend in solar cell efficiency based on black phase CsPbI_3_. Finally, we provide perspectives for future research related to the quest for optimum device efficiency and green energy.

## Abstract (web summary)

Black phase CsPbI_3_ easily transforms into the non-perovskite yellow phase, while losing the outstanding optoelectronic properties. In this review, the origin of the phase stability in CsPbI_3_ and strategies to stabilize the black phases exhibiting the α-phase or the relatively easily stabilized β/γ-phases are extensively discussed. Furthermore, a profound analysis of the CsPbI_3_ stabilization progress and the evolution of the performance efficiency records of black phase CsPbI_3_ is provided. Lastly, a prospective on future research on CsPbI_3_ solar cells pinpoints the current challenges and directs future research approaches toward more efficient and stable devices.

## Graphical abstract

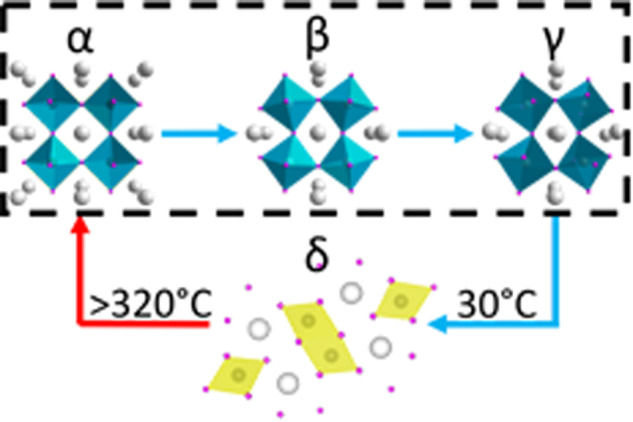

## Introduction

**Fig. 1:**
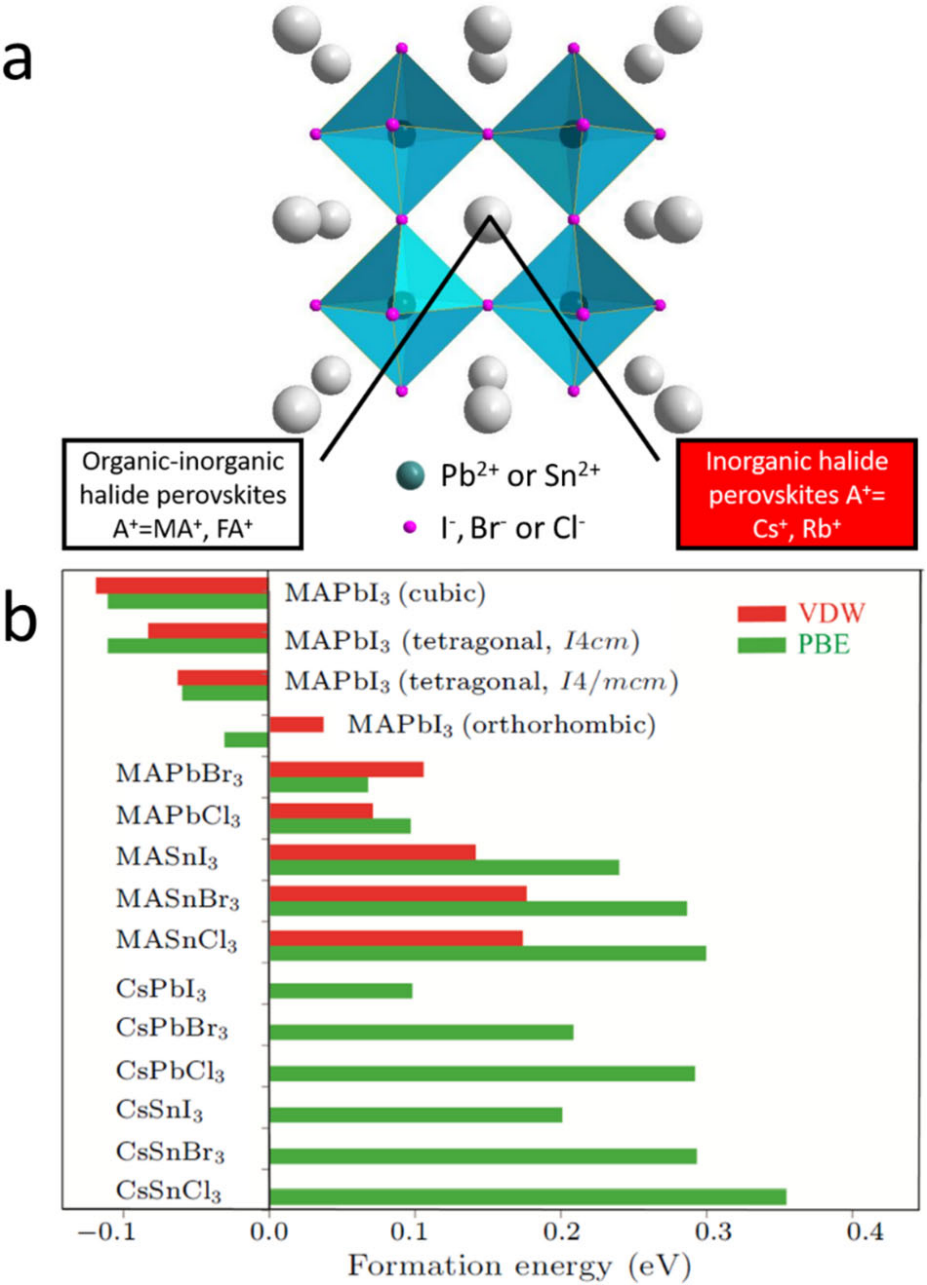
Crystal structures of 3D ABX_3_ perovskites and calculated energy cost for phase separation of ABX_3_. **a** Schematic overview of the typical ABX_3_ crystal structure for halide perovskites. The gray, black and pink spheres represent A cations (MA^+^, FA^+^, Cs^+^, and Rb^+^), B cations (Pb^2+^ or Sn^2+^), and X halides (Cl^−^, Br^−^, and I^−^), respectively. **b** Energy cost for phase separation of ABX_3_ calculated with the Perdew–Burke–Ernzerhof (PBE) and van der Waals (vdW) exchange–correlation functionals^8^. A positive value indicates that this composite is stable at T = 0 K. Reprinted with permission from ref. ^8^. Copyright 2018 IOP Science.

In recent years, lead halide perovskites (LHPs) have emerged as promising materials for photovoltaic (PV) and electroluminescence applications. In addition to their inexpensive and easy solution-processing characteristics, LHPs exhibit outstanding optoelectronic properties, such as broadband absorption, tunable bandgaps, long charge carrier diffusion lengths, and high defect tolerance, which enable high-performance solar cells^1^. In fact, the power conversion efficiencies (PCE) of LHP solar cells have already reached 26.1%^2^, which is close to the theoretical limit of 31%^3^. According to the “Shockley–Queisser (S-Q) triangle”^4^, these thermodynamic efficiency limits are mainly determined by the bandgap of the material, which can be measured via UV‒vis to infrared spectroscopy. To date, the most widely studied LHPs have three-dimensional (3D) structures with the formula ABX_3_, where A is a monovalent cation such as methylammonium (MA^+^), formamidinium (FA^+^) or cesium; B is a divalent cation such as Pb^2+^ or Sn^2+^; and X is a halide ion such as Cl^−^, Br^−^ or I^−^. Figure 1a shows the crystal structure of a 3D ABX_3_ compound. Goldschmidt’s tolerance factor refers to a dimensionless number that measures the stability and distortion of the crystal structure. Although it was originally developed to describe the structural stabilities of oxide perovskites, the tolerance factor is also valid for lead halide perovskites^5^. It can be calculated and used to evaluate the compatibility of a certain ion in a crystal structure. Goldschmidt’s tolerance factor (t) is expressed as follows:

$$t=\frac{{r}_{A}+{r}_{X}}{\sqrt{2}({r}_{B}+{r}_{X})}$$

where r_A_, r_B,_ and r_X_ are the ionic radii of the A-cation, B-cation, and X-anion, respectively. When *t* is larger than 1.0 or smaller than 0.71, a nonperovskite structure is favored. When the material has a *t* of 0.9-1.0, an ideal cubic structure will form. In addition to *t*, the octahedral factor *μ* is another important parameter and is defined as

$${\mu }={r}_{B}/{r}_{X}$$

where r_B_ and r_X_ are the ionic radii of the B-cation and X-anion, respectively. Stable BX_6_ octahedra are likely to form when *μ* is between 0.4 and 0.9. Several B and X ion combinations can form BX_6_ octahedra, which include Pb^2+^, I^−^, Br^−^ and Cl^−^.

However, recent studies have shown that the accuracy of *t* is insufficient for predicting perovskite stability^6^. This is because *t* correctly distinguishes between perovskite and nonperovskite materials for only 74% of perovskite materials. Thus, a new tolerance factor (τ) was developed^6^ with the form

$${\rm{\tau }}=\frac{1}{\mu }-{n}_{A}({n}_{A}-\frac{{r}_{A}/{r}_{B}}{\mathrm{ln}({r}_{A}/{r}_{B})})$$

where *n*_A_ is the oxidation state of the A cation, *μ* is the octahedral factor, r_A_, r_B_, and r_X_ are the ionic radii of the A-cation, B-cation and X-anion (r_A _> r_B_ by definition), respectively, and τ < 4.18 indicates a stable perovskite structure. This revised tolerance factor combines both Goldschmidt’s tolerance factor *t* and the octahedral factor *μ*, providing a more reliable tolerance factor for predicting perovskite stability.

The most popular 3D LHPs can be roughly divided into two types, namely, organic‒inorganic perovskites and pure inorganic perovskites, depending on the chemical nature of the A-cation, as shown in Fig. 1a. For example, methylammonium lead halides (CH_3_NH_3_PbX_3_, MAPbX_3_) are organic‒inorganic perovskites, and cesium lead halide (CsPbX_3_) is a pure inorganic perovskite. Inorganic perovskites contain only a symmetric and spherical cation, whereas in organic‒inorganic perovskites, the polar MA^+^-cation can vary in orientation at specific sites in the structure. Although the materials with the best performance are currently organic‒inorganic perovskites, the corresponding perovskite solar cells still suffer from low thermal stabilities due to the volatile natures of the organic MA and FA-cation, which, in turn, had stimulated further research on inorganic LHPs. In addition, for non-PV applications, such as light-emitting diodes and photodetectors, inorganic perovskites are more promising because of their stable optical properties under external stimuli^7^. Despite considerable research on inorganic LHP materials, particularly CsPbI_3_, the phase stability remains an important issue due to the critical revised *τ* values. Therefore, understanding and improving the phase stability of CsPbI_3_, which is vital for practical application, e.g., as an absorbing layer in solar cells, are highly important.

In this review, we discuss the nature of the stability and instability of CsPbI_3_ in terms of its chemical and structural origins. We also summarize recent state-of-the-art efforts in stabilizing the cubic (α) phase and pseudocubic (tetragonal β and orthorhombic γ with tilted octahedral structure) phases, including doping, applying additives, or new precursors. Furthermore, we evaluate the current state of solar cell efficiencies and reveal the relationship between phase stabilization and defect passivation. Finally, we provide perspectives for future research related to the quest for optimal inorganic LHP photovoltaic device efficiency and green energy.

## The origin of LHP instability

Theoretical predictions and experimental results have explicitly shown the instabilities of organic‒inorganic perovskites^8,9^. The low energy cost for decomposition of organic‒inorganic perovskites is the reason for the intrinsic thermodynamic instability associated with decomposition (see Fig. 1b), especially in ambient air^9^. For example, this decomposition process could be accelerated under a moist atmosphere^10,11^. Theoretical calculations by Zhang et al. showed that the decomposition process of MAPbI_3_

$${{\rm{MAPbI}}}_{3}\to {\rm{MAI}}+{{\rm{PbI}}}_{2}$$

is exothermic (at 0 K and zero pressure)^8,12^. Furthermore, according to the calculated vibrational free energies of LHPs as a function of temperature, the volatile nature of the organic cation MA^+^ is the main reason^8,12^ for perovskite instability due to the higher configurational entropy of MAI in comparison to that of MAPbI_3_. Substitution of the A-cation with inorganic ions can increase the energy cost of decomposition and thus increase the chemical stability. For example, if the A-site is occupied by Cs^+^, the decomposition energy is much greater than those of the organic‒inorganic counterparts, suggesting more stable compounds. Additionally, the configuration entropies of the constituent ions are much less significant in inorganic LHPs. Moreover, the chemical stabilities of inorganic LHPs are greater than those of organic‒inorganic LHPs. Aristidou et al. ^13,14^ reported that MAPbI_3_ degradation under light and oxygen was started by the reaction of superoxide with the MA^+^ moiety^15^.

**Fig. 2:**
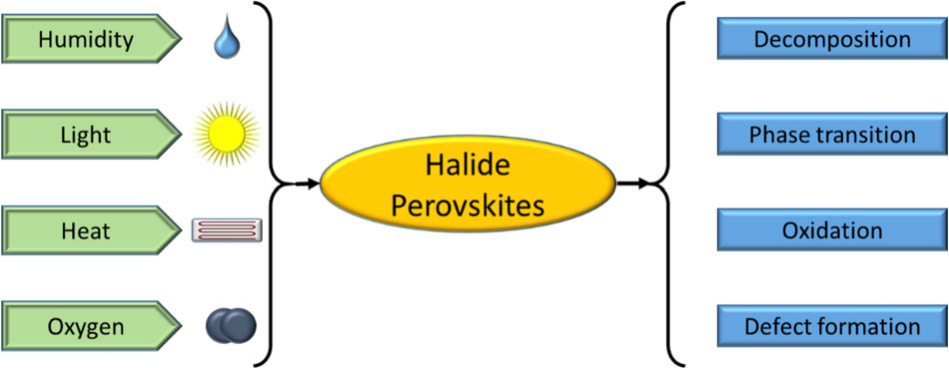
Degradation mechanisms. Mechanisms for halide perovskite degradation by humidity, light, heat, and oxygen.

Organic‒inorganic and fully inorganic lead iodide perovskites have their advantages and disadvantages. For example, organic‒inorganic perovskites are more easily processed in solution than inorganic perovskites and exhibit a more stable crystalline phase at room temperature. This is the reason why the highest efficiency of a perovskite solar cell is currently set by an organic‒inorganic derivative. However, the chemical stability is questionable^16^. Figure 2 provides a summary of the various degradation mechanisms for both organic‒inorganic and pure inorganic perovskites based on their chemical and structural phase stabilities, including phase transitions, decomposition, oxidation and defect formation. The structural stabilities of pure inorganic iodide perovskites must be addressed. The organic‒inorganic halide system is mainly chemical in nature^17–19^. For example, under high humidity or heat, organic‒inorganic halide perovskites exhibit poor chemical stabilities with almost instantaneous decomposition^20,21^. In contrast, inorganic iodide perovskites exhibit improved chemical stabilities but poor structural stabilities^20,21^.

**Fig. 3:**
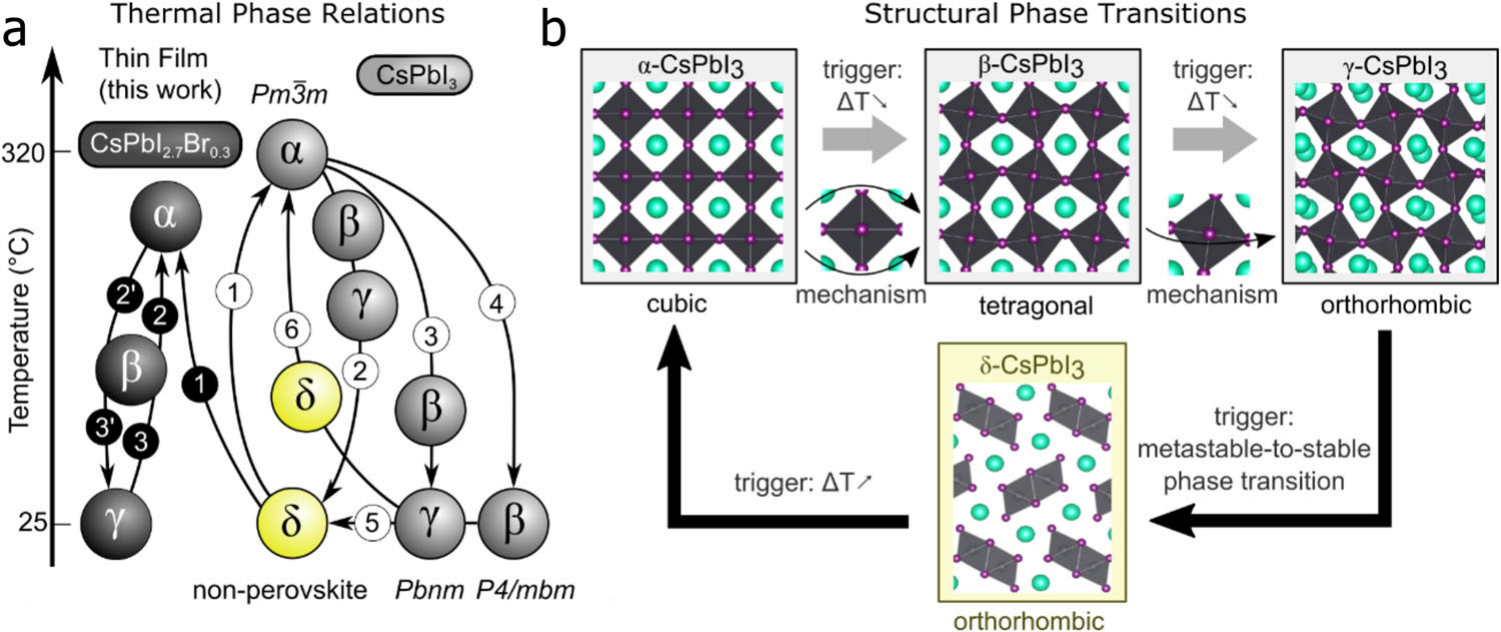
Phase transitions of CsPbI_3_. **a** Thermal phase relations and **b** schematic overview of the black α, β, and γ phases of inorganic CsPbI_3_^28^. Reprinted with permission from ref. ^28^. Copyright 2019 The American Association for the Advancement of Science.

The aforementioned factors demonstrate why inorganic LHPs are preferred over organic‒inorganic perovskites for stabilizing more materials. Among the inorganic perovskites, CsPbCl_3_ (2.7 eV) and CsPbBr_3_ (2.3 eV) exhibit 3D orthorhombic perovskite phases at room temperature. In principle, the calculated tolerance factors (t) for CsPbCl_3_ and CsPbBr_3_ are 0.82 and 0.81, respectively, which are not in the range of stable perovskites. The lower formation energy of the orthorhombic phase provides a stable perovskite structure at room temperature for these compositions^22^. Compared to CsPbI_3_, these two materials exhibit greater tolerance to external stimuli, such as humidity and heat, due to the stronger bonding between Pb and Cl/Br ions. Hence, owing to their excellent stabilities toward external stimuli, CsPbCl_3_ and CsPbBr_3_ are widely used in catalytic, laser, and LED applications. However, the large bandgaps of these materials significantly reduce long-wavelength absorption for PV applications. On the other hand, black phase CsPbI_3_ exhibits a small bandgap of ~1.7 eV with favorable optical properties for photovoltaic applications, such as UV to near-infrared absorptions and long carrier lifetimes. Once converted to the nonperovskite yellow phase, the outstanding optical and electronic properties of CsPbI_3_ materials disappear with the generation of a wide bandgap combined with poor optoelectronic properties. Unfortunately, the low *t* (*t* = 0.8) or high τ (*τ* = 4.99), based on the radii of Cs^+^, Pb^2+^, and I^−^, make the α-CsPbI_3_ structure unstable at room temperature. Black α-CsPbI_3_ is only formed at 330 °C and above. Upon cooling, CsPbI_3_ undergoes a complicated phase transition, as indicated in Fig. 3. With decreasing temperature, all three α, β, and γ phases are distinguished as photoactive black phases with slightly different band gap energies of 1.73 eV, 1.68 eV, and 1.75 eV, respectively^23,24^. In situ temperature-dependent X-ray diffraction studies have shown that CsPbI_3_ undergoes a transition from the α to the β phase and then to the γ phase at 281 °C and 184 °C, respectively^25^. When exposed to moisture^26^ or mild reheating^27^, γ- CsPbI_3_ is quickly transformed into the undesirable nonperovskite orthorhombic δ-phase^25^, which exhibits a large bandgap and poor electronic transport. Phase stabilization of the black phase CsPbI_3_ is, therefore, very important for use in photovoltaics. The main extrinsic factors that trigger the phase instability include temperature and moisture^28^. For example, metastable black β/γ CsPbI_3_ phases were kinetically trapped by thermal quenching to room temperature. The spontaneous strain from the unit cell shape changed during the phase transition, increased the energy penalty for restructuring and ultimately trapped the black β/γ CsPbI_3_ phases^28^. However, the yellow δ-CsPbI_3_ phase undergoes strain release once its saddle point is surmounted with mild reheating (60–100 °C)^28^. The α-phase CsPbI_3_ also quickly degrades to the nonperovskite yellow phase when exposed to a 33% RH nitrogen atmosphere at 23 °C for 75 min^27^. The β/γ CsPbI_3_ phases are also vulnerable to moisture attack, and they quickly destabilize and turning yellow when exposed^28^. This phase transition is induced by moisture and can be reversed back to the α-phase by reheating at 330 °C. It has been reported that water can be adsorbed on the CsPbI_3_ perovskite surface and act as catalysts to trigger the α to δ phase transition by forming halide vacancies and decreasing the free energy barrier for interconversion between the two phases^10,11,20,26^. In particular, due to the large solvation enthalpies of halide ions, the concentrations of halide vacancies increase significantly^10,11^. Compared with organic‒inorganic LHPs, CsPbI_3_ is relatively stable under illumination and current injection. However, phase segregation of Br-doped CsPbI_3_ caused by strong light and high currents is inevitable, as indicated by the presence of I-rich and Br-rich regions in perovskite materials^21,29^.

## CsPbI_3_ black phase stabilization

### Stabilization of the cubic α- phase

**Fig. 4:**
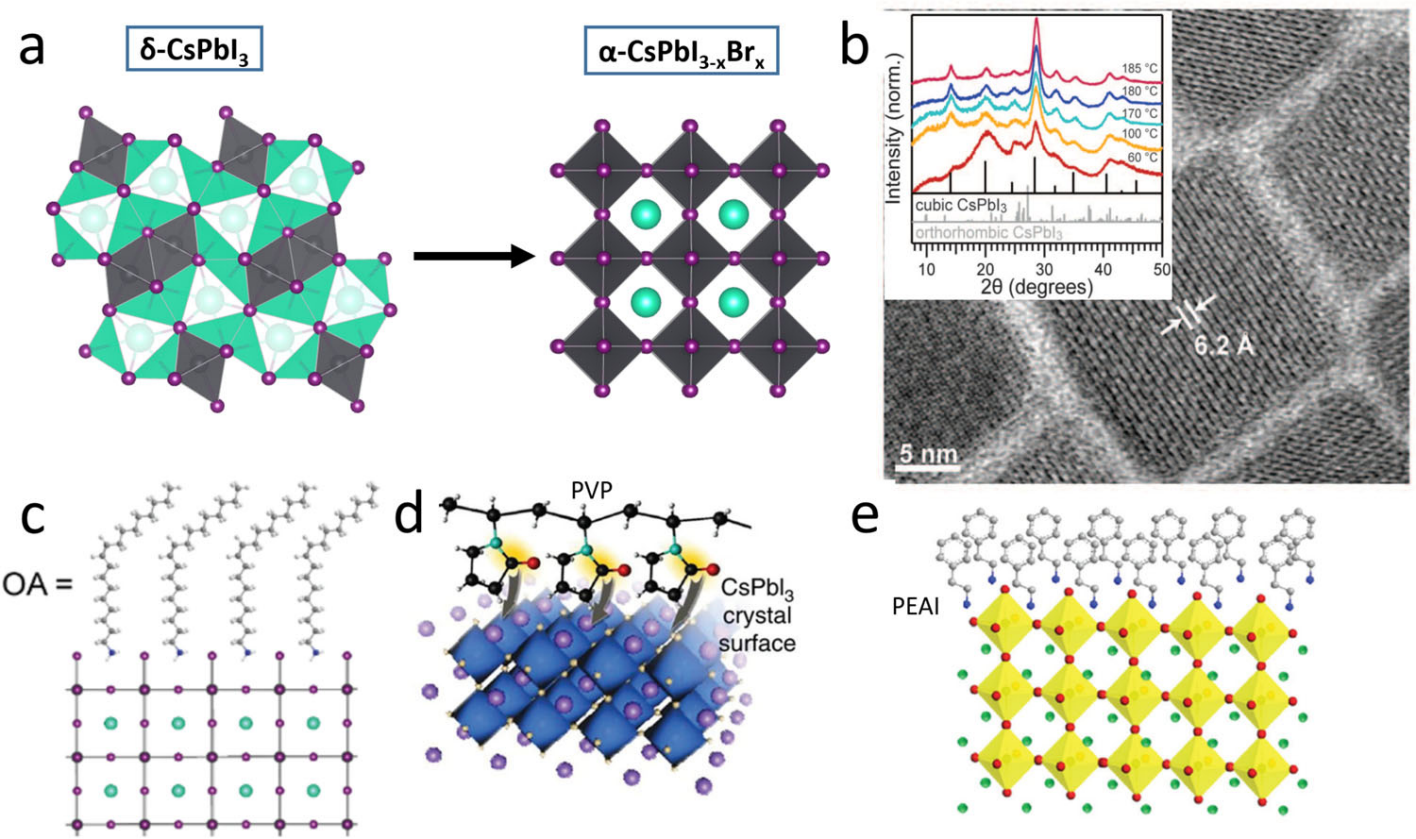
Stabilization of the CsPbI_3_ cubic (α) phase. **a** Schematic illustration showing stabilization of CsPbI_3_ via X-site (e.g., Br) doping. **b** TEM image and XRD patterns of colloidal α-CsPbI_3_ nanocrystals after storage under ambient conditions for two months^72^. Reprinted with permission from Ref. ^72^. Copyright 2016 The American Association for the Advancement of Science. **c** Oleic acid-stabilized α-CsPbI_3_^54^. Reprinted with permission from ref. ^54^. Copyright 2017 American Chemical Society. **d** Stabilization of α-CsPbI_3_ perovskite films with polyvinylpyrrolidone^55^. Reprinted with permission from ref. ^55^. Copyright 2018 Springer Nature. **e** Cubic phase CsPbI_3_ perovskite thin film with PEAI surface functionalization^66^. Reprinted with permission from ref. ^66^. Copyright 2018 Elsevier.

Although stabilizing the cubic CsPbI_3_ α-phase, which requires high-temperature annealing and broadening of the bandgap after doping, remains very challenging, great effort has been devoted to improving the doping/alloying, size engineering (Fig. 4b), additive, and surface functionalization (Fig. 4c–e) of the material.

#### α-Phase stabilization by doping/alloying

Doping/alloying is a typical strategy for tuning the electrical, optical, and magnetic properties of perovskite materials^30,31^. Typically, doping/alloying has been used to tune the tolerance factors of inorganic LHPs, as shown in Fig. 4a. According to the equation for the tolerance factor, a larger A and smaller B and X doping ions stabilize the cubic phase of CsPbI_3_.

##### A-site doping

The low *t* or high *τ* of CsPbI_3_ is mainly attributed to the small radius of the Cs ion. In this context, the tolerance factor *t*/*τ* can be increased or decreased by the incorporation of new ions with larger radii, which results in a greater average radius for the A-site ions. For example, the larger ionic radii of MA^+^ and FA^+^ beneficially impact the *t*/*τ* value and stabilize the structure. However, the incorporation of an organic cation decreases the thermal stability. To date, no suitable inorganic cation has been identified to increase *t* or decrease *τ* for the CsPbI_3_ compound because cesium is the largest (nontoxic) Group I element. As mentioned, Cs is too small to support a PbI_6_ corner-sharing framework, and smaller alkali elements, such as K^+^, Na^+^, or Rb^+^, only exacerbate this problem. However, as anticipated, the introduction of relatively smaller K^+^^32^, Rb^+^^33^, and Na^+^^34^ cations into the A sites of CsPbI_3_ results in improved phase stabilities under ambient conditions. This is believed to result from contraction of the PbX_6_ octahedral volume^35^. In addition, doping with smaller A sites, such as K^+^, enhances charge carrier transport, thereby improving the solar cell performance (efficiency = 10%)^32^.

##### B site doping

Changing the B site is a great challenge because the octahedral factor μ and anion sublattice can be affected simultaneously. Doping with smaller B doping ions is preferred due to the tolerance factor equation^35^. Recently, several nontoxic metals with small radii, such as Ca, Mn, Sn, Bi, and Sb, have been investigated as partial lead replacements^36–40^. Some metals, including Ca^39^, Mn^40^, and Sn^38^, only changed the composition without altering the requirement for high-temperature annealing to achieve the α-phase. Due to the large difference in sizes between Ca^2+^/Mn^2+^ and Pb^2+^, only a slight amount of Ca^2+^/Mn^2+^ can be doped into CsPbI_3_^39,40^.

Upon substituting lead with Ca^2+^ ( ~ 5%), the CsPbI_3_ cubic phase became more stable after annealing at 300 °C, and the film quality improved^39^. Ca^2+^ incorporation resulted in larger grains and smoother films by reducing the colloidal particle sizes in the precursor solution. A Ca-rich oxide layer was formed at the surface and exhibited a passivation effect, resulting in a longer carrier lifetime. In solar cell devices, 5% of the lead was replaced by Ca^2+^, and the resulting device exhibited a 13.5% efficiency. However, only a few Ca^2+^ ion can be doped into the lattice of CsPbI_3_ because most of the Ca^2+^ forms a Ca-rich oxide layer at the surface^39^. Like Ca^2+^, only a slight amount of Mn^2+^ can be doped into CsPbI_3_^40^. According to DFT calculations, unlike the broader bandgap observed after Ca^2+^ doping, the Mn^2+^ dopant energy levels are located in the conduction band^40^; therefore, their effect on the absorption spectra of stabilized CsPbI_3_ thin films is negligible. Compared to Mn and Ca, Sn is a perfect candidate for doping Pb sites because Sn^2+^ is only slightly smaller than Pb^2+^ and easily forms an appropriate *t*^41^. Although CsPb_1-x_Sn_x_I_3_ exhibits high phase stability with a broad absorption at close to infrared wavelengths^42^, the oxidation of Sn^2+^ to Sn^4+^ remains a point of concern^43^. Moreover, upon doping with trivalent metals such as Bi^3+^^37^ and Sb^3+^^36^, the black phase can be formed at temperatures close to 100 °C. Unlike the divalent Ca^2+^ and Mn^2+^, doping with trivalent Bi^3+^ and Sb^3+^ normally reduces the crystal dimensionality from 3D to 1D by forming an impurity phase. However, it has been found that precisely controlling the Bi^3+^ component enabled a transformation from the δ-phase to the α-phase with 4 mol% Bi^3+^ incorporation in the CsPbI_3_ lattice^37^.

##### X site doping

Another way to tune the *t* value is to substitute some of the I^−^ with Br^−^ or Cl^−^, which have smaller radii, leading to stable perovskite structures. A prominent example is the CsPbI_3-x_Br_x_ alloy^21,44^. Considerable effort has been focused on substituting some of the I ions with the smaller Br ions to decrease the average radii of the X-site ions while retaining the all-inorganic composition and structure. Snaith et al. fabricated a series of CsPb(I_1-x_Br_x_)_3_ materials and reported a 9.8% PCE for CsPbI_2_Br^45^. When isopropanol was combined with an antisolvent, further improvement in the CsPbI_2_Br efficiency of 16.07% was achieved, as indicated by larger grain sizes and excellent long-term moisture stabiliies^46^. Although a more stable structure with a slightly distorted perovskite structure was obtained by Br doping, the undesired blueshift of the absorption edge due to the enlarged bandgap remains a point of concern since the bandgap of CsPbI_3_ (~1.7 eV) is already relatively large for a single-junction active layer. Furthermore, Br doping avoids the phase separation problem by forming a Br-rich and an I-rich phase under continuous irradiation^21^ and thermal annealing^47^.

Some work has been done on stabilizing the cubic phase with high Cl doping levels^10^. However, unlike that of Br doping^25^, the effect of Cl doping on the intrinsic phase transition behavior still needs further investigation. As with Ca^2+^ and Mn^2+^ doping, the widely accepted reason is the size difference between chloride and iodide^48^. However, a good way to tune the tolerance factor is by substituting some of the Cl in the stable perovskite CsPbI_3_ with I. However, for the closely related MAPbI_3_, the extent of chloride incorporation into the MAPbI_3_ phase is still debated^49^. Despite widespread interest in the properties of the MAPbI_3-x_Cl_x_ mixed crystals, chloride appears to have limited miscibility with iodide perovskites^10,50^. The miscibility of chloride in CsPbI_3_ also faces the same issues. As a direct result, Cl cannot be incorporated into CsPbI_3_. It has been reported that the CsPbI_3-x_Cl_x_ majority phase only forms a mole fraction of approximately 2%^10^. This appears to be an upper limit for the extent of chloride incorporation in the prevailing iodide lattice.

A codoping strategy has been proposed to overcome the undesired blueshift of the absorption edge. Codoping of Br and In in CsPbI_3_ generated excellent stability in air while retaining the relatively low bandgap energy^51^. Moreover, these codoping methods have been found to suppress Pb-related intrinsic defects, possibly due to stronger bonding between In and the halide atoms. The development of new strategies to increase the inorganic LHP phase stability without increasing the bandgap energy is critical for improving the efficiencies of PV devices. Within this context, the multielement doping strategy^51^ constitutes a promising avenue for further stabilization of inorganic LHPs and their related devices.

#### α-Phase stabilization via additives

An alternative method for stabilizing the α-phase is to incorporate suitable nonvolatile additives (as shown in Fig. 4c) into the CsPbI_3_ solution, as both the surface energy and the grain sizes can be tailored with nanolayers encapsulating the CsPbI_3_ perovskite grains. The types of additives can be roughly divided into long-chain ammonium cations, polymers (e.g., polyethylene oxide (PEO)^52^), and large polar organic molecules (e.g., sulfobetaine^53^ zwitterions). This is because longer carbon chains prevent grain growth and aggregation and generate smaller grain sizes^54^. Stabilization of the black phase requires strong interactions between the additive and as-crystallized CsPbI_3_ (e.g., oleylammonium^54^ (OA^+^) ~ 1.7 nm). Additives can be directly introduced into perovskite solutions with a one-step deposition method. Through the combination of coordinate bonding or ionic bonding between additives and perovskite materials, the incorporated additives ultimately decrease the surface energy, reduce the grain sizes, and form nanoscale encapsulation layers on the CsPbI_3_ grain surfaces^55^. For example, incorporation of the polymer polyvinylpyrrolidone (PVP) into the CsPbI_3_ precursor induced surface passivation and stabilized the cubic phase of CsPbI_3_ by reducing the surface tension^55^. It was proposed that the electron density on the CsPbI_3_ surface was increased by chemical bonding between the amide groups in PVP and CsPbI_3_^55^. This interaction ultimately reduced the surface energy and improved the phase stability of CsPbI_3_.

Hydroiodic acid (HI) or dimethylammonium iodide (DMAI) may be useful for stabilizing the black phase CsPbI_3_, although the underlying mechanism is not fully understood. Recently, almost every major breakthrough in the efficiencies of CsPbI_3_ solar cells has been related to these additives. Snaith et al. stated that HI is a favorable additive because the addition of HI does not obviously change the bandgap energy of CsPbI_3_^56^. Hao et al. and Xiang et al. reported that HI reduces the annealing temperature by inducing tensile lattice strain, which contributes to phase stability^57,58^. Next, Long and coworkers reported that HI reacts with PbI_2_ in DMF to produce HPbI_3_^59^. Finally, hydrogen lead iodide (HPbI_3_), obtained from the reaction of PbI_2_ with HI in DMF, has been extensively used to replace PbI_2_ in the synthesis of phase-stable CsPbI_3_^57^. HPbI_3_ can be used instead of PbI_2_ to stabilize the α-phase of CsPbI_3_ at room temperature and stretch the lattice to generate a broader bandgap^57^. Although the addition of HPbI_3_ made significant progress in improving the conversion efficiency, the underlying mechanism for stabilizing the CsPbI_3_ black phase has not been determined. Even the existence of HPbI_3_ is questionable based on the opinion that HI cannot be complexed with a metal or that HPbI_3_ readily dissociates into PbI_2_ and HI. Hence, Ke et al. claimed^60^ that HPbI_3_ does not exist and that DMAI was formed by the reaction of HI with DMF and was incorporated into the perovskite structure as a dopant, thereby optimizing the tolerance factor^60^. Marshall et al. quantified the effect and limitation of DMA^+^ replacing Cs^+^ and reported that stable α-phase CsPbI_3_ was only formed via 25% replacement of Cs^+^ with DMA^+^^61^. Upon increasing the concentration of DMA, phase separation of CsPbI_3_ and DMAPbI_3_ occurred^61^. Recently, Wang et al. proposed a new point of view indicating that DMAI cannot be doped into the CsPbI_3_ lattice because of its volatile nature^62^. They found that DMAI started to sublime at 210 °C without leaving a DMAI residue in the final CsPbI_3_ perovskite film^62^. Until recently, the existence of HPbI_3_, and the exact mechanism for formation, are not fully understood.

#### α-Phase stabilization via surface functionalization

Surface functionalization (shown in Fig. 4e) is commonly used in stabilizing the organic‒inorganic LHP phase^63^. For example, pure FAPbI_3_ with cubic symmetry was stabilized by posttreatment with long-chain alkyl or aromatic ammonium cations^63^. As with additives, surface functionalization decreased the surface formation energy of perovskite crystals through chemical bonding and also stabilized the cubic phase^63^. The only difference between additives and surface functionalization is the deposition method used^64^. In contrast to the direct introduction of additives into precursor solutions, surface functionalization methods normally involve antisolvent or posttreatment processes^64^. The result is that the grain sizes are not tailored via surface functionalization. Surface posttreatment with the formation of a thin functionalization layer can also be used to stabilize CsPbI_3_ perovskite thin films^65^. When a layer of PEAI was deposited on top of a CsPbI_3_ thin film, the alkylammonium cations replaced the surface Cs^+^ cations^65^ and simultaneously formed a hydrophobic barrier,^66^ which protected CsPbI_3_ against moisture-induced degradation and stabilized the phase. In addition, PEA^+^ on the CsPbI_3_ crystal surface also caused surface passivation^66^. Subsequently, PEABr was used to functionalize the film surface, which led to Br-doped thin films with PCEs of 16.3%^67^. Approximately 91% of the initial PCE was retained after 500 h of operation. In addition to PEA^+^ cations, other organic cations, such as diethylenetriamine iodide (DETAI_3_), may also stabilize and/or passivate CsPbI_3_^68^. Compared with monoamine cations, DETA^3+^, a polyamine, is superior for stabilizing the perovskite phase^68^. These molecules with multiple amino groups cross-link the CsPbI_3_ units with the adjacent units on the grain surface, preventing octahedral tilting and suppressing the phase transition.

#### α-Phase stabilization via dimensionality engineering

Upon decreasing the material dimensions, new properties, such as enhanced thermal stability, can emerge^69,70^. The bulk α phase of CsPbI_3_ is not stable at room temperature, while CsPbI_3_ nanocrystals prepared by solution processing exhibited greater phase stability due to their size confinement, as shown in Fig. 4b. Kovalenko et al. reported that α-CsPbI_3_ nanocrystals measuring 100 to 200 nm in length were quickly transformed into the δ phase, while α-CsPbI_3_ nanocrystals measuring 4 to 15 nm in length remained in the α phase for one month at room temperature^71^. Subsequently, Swarnkar et al. demonstrated that perovskite solar cells made of phase-stabilized CsPbI_3_ nanocrystals exhibited high efficiencies of 10.77%^72^. A lower surface energy or high surface strain of the nanocrystals could have caused phase stabilization^35,73^, although the exact origin of this phenomenon remains to be elucidated. For example, when the energy contribution from the surface outweighed that from the bulk, as the sizes of crystallites decreased to the nanoscale, the cubic phase became stable at room temperature. Furthermore, the capping ligands on the surface protected the nanocrystals from segregation or aggregation into large crystals. However, the ligands on the perovskite surface may block efficient transfer of the charge carriers. In a later study, a posttreatment strategy using organic halide salts (such as FAX) was shown to control the CsPbI_3_ nanocrystalline morphology while enhancing electronic coupling between neighboring nanocrystals, which provided a solar cell device with a certified PCE of 13.43%^74^. To improve the stability of the α-phase further, CaF_2_ was incorporated into the crystal lattice of CsPbI_3_ nanocrystals, resulting in passivation of lattice defects^75^. Some work has been done on ligand-assisted syntheses of α-CsPbI_3_ perovskite nanocrystals. Compared with those of the standard structure, the smaller lattice constant and stronger bonding between the Pb and I atoms in the octahedra were observed^76,77^. It was concluded that the smaller lattice constant of the CsPbI_3_ nanocrystals resulted in higher phase stability under ambient conditions^76,77^. In addition, defect passivation of stabilized CsPbI_3_ nanocrystals will be the focus of future research intended to improve the performance of CsPbI_3_ nanocrystal-based electronic devices. For example, surface defects of CsPbI_3_ nanocrystals can be controlled with thiolate surface passivation due to the peculiar binding of these materials with iodine vacancies^78^. Future attempts to understand ligand‒crystal interactions and the mechanism for phase stabilization with nanosized crystals will lead to more stable and efficient inorganic LHP-based LEDs and solar cells.

#### α-Phase stabilization via strain

The strain in semiconductors caused by lattice mismatches, thermal stress and external stimuli could eventually result in the formation of point defects and dislocations via strain relaxation^79,80^. Similarly, strain is inevitably present in LHPs and their films because of an intrinsic lattice mismatch or extrinsic mismatch at the interface (such as the thermal expansion coefficient mismatch between MHPs and the substrate^81^). The presence of point defects after releasing the strain is the main cause of degradation of LHP films^80,82^. However, a strain-inducing strategy has recently been shown to be a new way to stabilize the CsPbI_3_ cubic phase. As with the formation of stable CsPbI_3_ nanocrystals due to their lower surface energies, the surface formation energy can also be reduced under compressive strain^73^. Similarly, for CsPbI_3_ grown in vertically aligned anodized aluminum oxide nanopores, the magnitude of the microstrain was adjusted by changing the sizes of the pores^73^. This enabled fabrication of stable α-phase CsPbI_3_ by introducing strain. Although little attention has been paid to the strain method, it provides a promising avenue for stabilization of inorganic LHPs and related devices. For example, after Br doping, the α-phase of CsPbI_3_ was more stable after the introduction of interfacial strain. As strain originates from structural distortion, the atomic shifts resulting from a phase transition or from the interface due to differences in the thermal expansion coefficients can be evaluated with GIWAX measurements. Relative to the free-standing structure, the changes caused by tilting and distortion of the perovskite unit cell can be linked to the degree of strain. The interested reader can consult reference^35^ for a more in-depth discussion.

### Stabilization of β/γ-phase CsPbI_3_

**Fig. 5:**
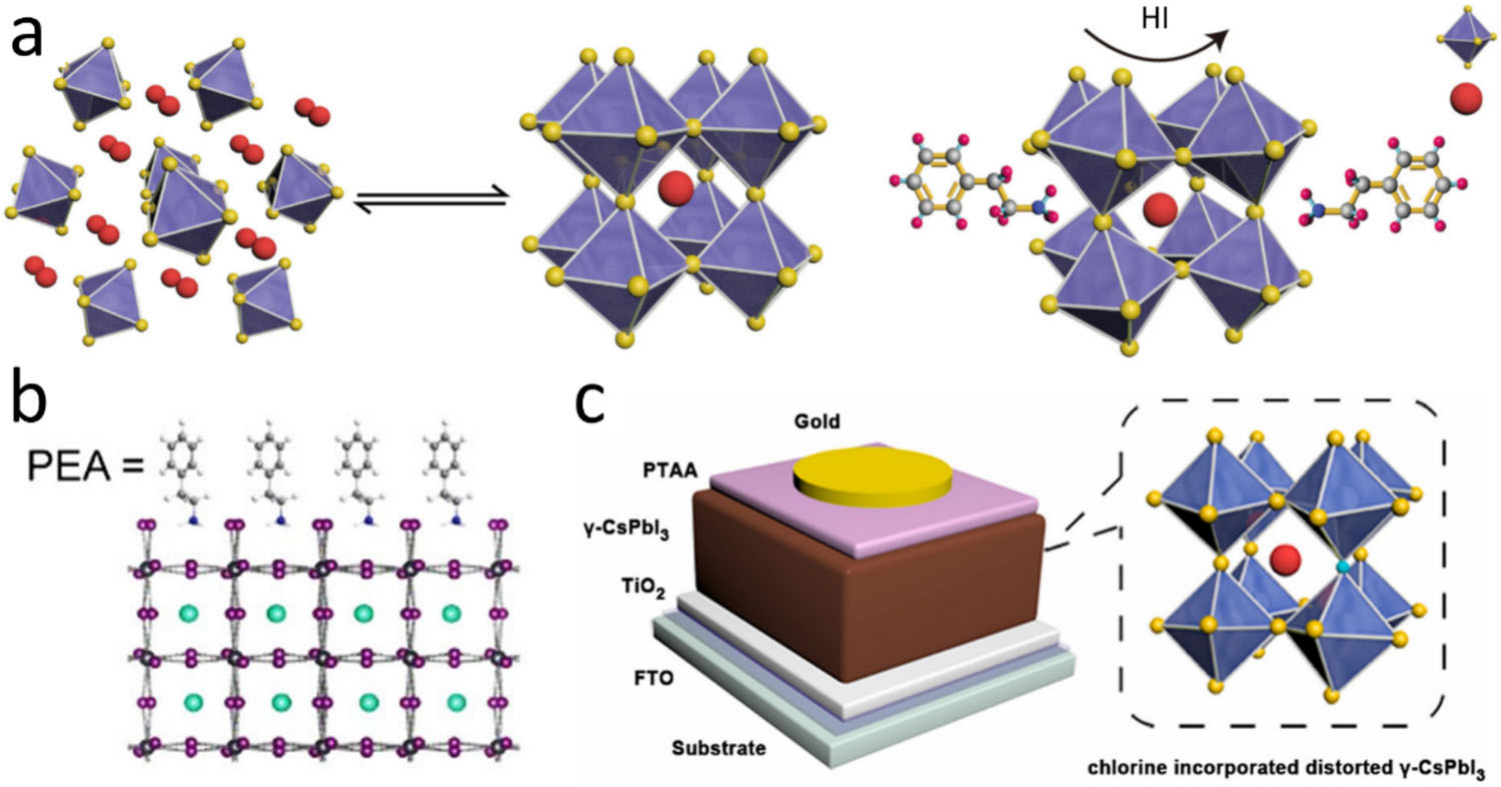
Stabilization of the CsPbI_3_ β/γ phases. **a** Synthesis of β/γ-CsPbI_3_ by incorporating HI/DMAI additives^115^. Reprinted with permission from ref. ^115^. Copyright 2018 Springer Nature. **b** PEA-stabilized CsPbI_3_ in the β phase^54^. Reprinted with permission from ref. ^54^. Copyright 2017 American Chemical Society. **c** Stabilization of γ-CsPbI_3_ via Cl doping^85^. Reprinted with permission from ref. ^55^. Copyright 2019 Elsevier.

Despite considerable effort, stabilizing the cubic CsPbI_3_ α-phase remains very challenging. For example, a high-temperature (> 300 °C) annealing procedure was used. This ultimately resulted in a higher resistance of the ITO substrate and limited usage of some efficient hole transport materials, such as poly(3,4-ethylenedioxythiophene) polystyrene sulfonate (PEDOT:PSS). Therefore, much attention has been given to stabilizing the β/γ-black phase. Compared with those of the pure α-phase, the β/γ-phases usually exhibit split diffraction peaks due to their tilted octahedral structures and similar bandgap energies. These two phases easily form at nearly room temperature (∼100 °C) and normally do not need high-temperature annealing because their formation energies^83^ are lower than that of the cubic phase. Several approaches have been used to obtain a stable β/γ-phase, including doping, HI and DMAI additives, or passivating organic ligands, as shown in Fig. 5.

#### β/γ-Phase stabilization by doping/alloying

As discussed in the previous section, tuning the composition by X-site doping and annealing above 300 °C seems to be a promising approach for increasing the stability of α-phase CsPbI_3_. Numerous attempts have been made at stabilizing the β/γ-phase of CsPbI_3_ by doping with Br and Cl. In contrast to high-temperature annealing for α-phase stabilization, Sanchez et al. reported that black γ-phase CsPbI_1.8_Br_1.2_ was accessed by annealing at 100 °C. Most impressive, a 90% efficiency was retained after heating the perovskite film at 200 °C for 1 hour. Chai et al. reported a novel CsPbBr_3_ seed method for fabricating β-phase CsPbI_3_Br_3-x_ with larger crystallite and grain sizes^84^. Bromide ion diffusion from the interface to the upper surface passivated the trap densities, improved the energy level alignment, and finally resulted in enhancement of the efficiency from 16.09% to 18.60%^84^. In addition, a γ-CsPbI_3_ film was obtained by the introduction of 3% Cl ions^85^. The performance and black phase stability after Cl doping were also enhanced by minimizing the trap density^85^. As with Cl-doped MAPbI_3_^78^, a lower trap density and greater carrier mobility were observed for γ-phase Cl-doped CsPbI_3_ because chloride doping neutralized the iodide-related traps^85,86^. B site doping, such as the incorporation of Mg^2+^, has also been used to stabilize γ-phase CsPbI_3_^87^. Mg alloying led to a decrease in the phase transition temperature to black γ-CsPbI_3_^87^. Additionally, some papers have reported a codoping strategy to stabilized β/γ-phase CsPbI_3_^88–90^. For example, codoping with Li^+^ and F^−^ was investigated for stabilizing β-phase CsPbI_3_ by increasing the grain sizes, improving the crystallinity, and reducing the defect density^91^. Similarly, codoping with In^3+^ and Br^−^ improved the crystal quality and thermal stability of β-CsPbI_2.5_Br_0.5_^92^. Even after heating at 100 °C for more than 1632 h, 80% of the initial efficiency was maintained^92^. Pansa-Ngat et al. also reported^93^ codoping with Ca^2+^ and Mn^2+^ to retain the γ-phase for up to 16 days under high humidities of 40–60%. A series of materials codoped with Eu^3+^, ln^3+^, Cd^2+^, and Tb^2+^ to stabilize γ-CsPbI_2_Br was reported by Mali et al. ^88–90^. It was concluded that codoping is an effective way to improve the conversion efficiency and stability in ambient air^88^.

#### β/γ-Phase stabilization with HI and DMAI additives

As with α-phase stabilization, the incorporation of additives is also a good way to stabilize the β/γ-phases of CsPbI_3_. The strong interactions of coordinate bonding or ionic bonding between additives and perovskite materials can result in distorted PbI_6_ octahedra and stabilize the β/γ phases of CsPbI_3_^55^. For example, by adding HI, Snaith et al. fabricated working γ-phase CsPbI_3_ solar cells in 2015^56^. They developed a low-temperature phase transition route at 100 °C. This occurred because size-driven effects are likely to increase the stabilities of smaller crystals, ultimately leading to a lower phase transition temperature^28^. It has been found that HI facilitated the formation of small crystals in films without changing the optical properties. Subsequently, a combined treatment of CsPbI_3_ PSCs with HI and isopropanol generated a PCE of 4.13%, as reported by Luo et al. ^94^ These authors demonstrated the formation of a stable CsPbI_3_ layer from the intermediate Cs_4_PbI_6_. Moreover, Hu et al. reported a one-step preparation method for an orthorhombic CsPbI_3_ thin film with HI and water additives^23^. Furthermore, added DMAI facilitated the formation of β-phase CsPbI_3_^24^. Finally, Wang et al. reported that the γ-phase was obtained with a 0.5–0.7 DMAI–to-Cs ratio, while the β-phase was formed when the DMAI–to-Cs ratio was 1:1.5^62^. Importantly, they also showed that DMAI was completely removed after annealing at 200 °C, confirming that DMAI was a volatile additive in the fabrication of CsPbI_3_ perovskites. Notably, the DMAI residues in CsPbI_3_ films could degrade the PSC performance and stability. However, the mechanism of action for DMAI, including manipulations of the strain, surface energy and chemical bonding, is still not fully understood. For example, although the β and γ phases can be obtained separately by adjusting the amount of additive used^62^, how the strain is manipulated is still unclear.

#### β/γ-Phase stabilization via surface functionalization

Complementary to the previous strategy, organic molecules can also stabilize black γ-phase CsPbI_3_ at low temperature. Compared with the organic ligands used in stabilizing the cubic phase, these organic molecules normally have relatively shorter carbon chains^54^. Taking oleylammonium (OA^+^, chain length of ~1.7 nm) and PEA^+^ (chain length of ~0.6 nm) as examples, two metastable CsPbI_3_ perovskite polycrystalline materials in the α and β phases were synthesized via one-step spin coating film deposition by using OA^+^ and PEA^+^, respectively^54^. The molecules for stabilizing β/γ-phase CsPbI_3_ included phenylethylammonium (PEA^+^)^54^ and ethylenediamine (EDA^+^)^95^. As in cubic phase stabilization methods, these organic ligands were generally introduced into perovskite precursor solutions or via antisolvent/posttreatment processes. With PEA^+^ used as a surface capping ligand, the γ-phase was stabilized at 120 °C and exhibited a PCE of 6.5%^54^. In addition to the carbon chain lengths, the mechanism for generating different phases via organic ligands could involve multiple factors, including the molecular forces between chains, the deposition method and the concentrations of organic molecules.

The intermolecular interactions between long carbon chains of linear molecules may also result in differences in phases and phase stabilities^96^. For example, the stability of PEA^+^ is greater than that of hexylammonium (HA) due to its narrower intermolecular spacing^96^. Direct PEA^+^ doping did not create any β/γ-phases of CsPbI_3_^97^. The introduction of a small amount of PEAI stabilized the β phase^54^ of CsPbI_3_; however, posttreatment with a high concentration of PEAI resulted in a stable cubic phase^66^.

#### β/γ-Phase stabilization via strain

As with α-phase CsPbI_3_, which was stabilized with strain, γ-phase CsPbI_3_ has been stabilized through the introduction of substrate interface strain^28^. It was found that the formation energies of these materials decreased under interfacial strain^28^. Zhu et al. reported the observation of gradient energy band bending induced by interface strain, which seriously affected the carrier transport properties and conversion efficiencies^98^. The strain-free device exhibited a reduced charge carrier lifetime and a higher efficiency with negligible hysteresis, suggesting better carrier extraction^98^. Similarly, they reported that decreased microstrain and a more ordered crystalline orientation dramatically improved the optoelectronic properties and light-heat stability^99^. Overall, strain is a double-edged sword for both phase stability and performance. Later, a photolithographic approach was developed to embed an interfacial PbI_2_ microstructure into a CsPbI_3_ perovskite film, causing vertical strain and eventually stabilizing the γ-phase^100^. The black γ-phase induced by vertical strain demonstrated long-term stability beyond 2.5 years in a dry atmosphere^100^. After applying this stabilized film, the performance stabilities of planar CsPbI_3_ perovskite photodetectors were improved beyond those of γ-phase CsPbI_3_ stabilized with interface strain. In addition, Bai et al. prepared a heterojunction structure with 0D Cs_4_PbI_6_ and 3D γ-CsPbI_3_ by adjusting the stoichiometric ratio of CsI to PbI_2_^101^. It was revealed that the Cs_4_PbI_6_ particles surrounding the CsPbI_3_ grains imposed spatial constraints on the 3D CsPbI_3_, leading to a decrease in the associated lattice constant and finally stabilizing the black γ-phase^101^.

## Relationship between phase stabilization and defect passivation

As defects have critical impacts on the functional properties of a semiconductor^102–104^, it is important to discuss the relationship between phase stabilization and defect passivation. With the stabilization processes discussed above, defect passivation can be simultaneously realized, as shown in Table 1. Despite the use of sophisticated techniques and strict control of the reaction conditions, the formation of high concentrations of native defects during LHP crystal growth could not be avoided^104^. Theoretical calculations and experiments assigned the LHP defects to shallow defect energy levels. However, deep traps, such as iodide interstitials and extrinsic surface defects, were present inside the bandgap. In this context, stabilizing the black phase and passivating the surface defects was essential and was achieved with a single processing method. As discussed above, doping/alloying is a good way to tune the tolerance factor and stabilize the CsPbI_3_ black phase. In most cases, higher PLQYs and longer PL lifetimes were also observed, implying lower trap densities in these doped composite films. Lau et al. reported that the surface defects of Ca-doped CsPbI_3_ films were passivated by the formation of a Ca-rich oxide layer^39^. Similarly, X-site doping, including Br and Cl doping, was used to tune the tolerance factors of CsPbI_3_, and suppressed trap densities were demonstrated by a longer PL lifetime in the doped thin films and higher open-circuit voltages in the solar cells^85,105^. This was particularly true for iodide-containing inorganic LHPs due to the stronger binding of lead with chloride and bromide^44,85,105^. The codoping strategy passivated multiple defects. For example, dual doping of Br^−^ and In^3+^ passivated I- and Pb-related defects due to the stronger bonding of In^3+^ and Br^−^ and their smaller ionic radii. Similarly, a low trap density of 4.35 × 10^15 ^cm^−3^ was achieved by codoping Zn^2+^ and Cl^−^ into CsPbI_3_ because of Pb- and I-related defect passivation^106^. Moreover, this strategy allows the implementation of microstrain at different locations in the perovskite structure, which can compensate for each other, thereby stabilizing the favored black phase^106^.

**Table 1:** Summary of progress on defect passivation with black phase CsPbI_3_.

Method to reveal trap density	Materials	Pristine trap density (cm^−3^)	Trap density after passivation(cm^−3^)	Passivating solutions	References
SCLC	α-CsPbI_3_	1.73 × 10^15^	2.49 × 10^14^	hexaoxacyclooctadecane ether surface passivation	^141^
TAS	γ-phase CsPbI_3-x_Br_x_	n_e_ = 1.29 × 10^16^ n_h_ = 1.83 × 10^15^	n_e_ = 1.17 × 10^16^ n_h_ = 1.78 × 10^15^	Br doping	^105^
SCLC	α-CsPbI_3_ QDs	1.9 × 10^12^	8.4 × 10^11^	2-Aminoethanethiol surface passivation	^142^
SCLC	γ-phase CsPbI_3_	2.02 × 10^16^	8.75 × 10^15^	Phenyl ligands surface passivation	^143^
SCLC	α-CsPbI_3_	3.7 × 10^15^	2.4 × 10^15^	(adamantan-1-yl) methanammonium Surface passivation	^144^
SCLC	γ-CsPbI_3_	9.2 × 10^15^	5.2 × 10^15^	(Adamantan-1-yl) Methanammonium surface passivation	^134^
SCLC	β-CsPbI_3_	n_e_ = 3.7 × 10^15^ n_h_ = 4.7 × 10^15^	n_e_ = 2.5 × 10^15^ n_h_ = 4.3×10^15^	Light soaking	^145^
SCLC	α-CsPbI_3_ QDs	1.93 × 10^16^	1.14 × 10^16^	Glycine surface passivation	^146^
SCLC	α-CsPbI_3_	2.7 × 10^16^	9.2 × 10^15^	YbCl_3_ additives surface passivation	^147^
SCLC	α-CsPbI_2_Br	9.57 × 10^16^	8.35 × 10^16^	Incorporation of polyethylene glycol	^121^
SCLC	α-CsPbI_3-x_Br_x_	n_e_ = 0.9 × 10^14^ n_h_ = 0.85 × 10^14^	n_e_ = 0.7 × 10^14^ n_h_ = 0.64 × 10^14^	GA_2_PbI_4_ surface passivation	^123^
SCLC	β-CsPbI_3-x_Br_x_	2.25 × 10^15^	4.76 × 10^14^	DTABr passivation	^124^
SCLC	α-CsPbI_3_	n_e_ = 9.71×10^15^	n_e_ = 4.04 × 10^15^	Ti_3_C_2_F_x_ QDS interface passivation	^125^
SCLC	α-CsPbI_3_	n_e_ = 4.25×10^15^	n_e_ = 1.62 × 10^15^	Zwitterion salt passivation	^110^
SCLC	γ-CsPbI_3_	n_e_ = 1.79×10^15^	n_e_ = 9.63 × 10^14^	Vacuum thermal annealing	^148^
SCLC	γ-CsPbI_3_ (NCs)	n_e_ = 4.54×10^15^	n_e_ = 3.61 × 10^15^	GA_2_CO_3_ surface passivation	^130^
SCLC	γ-CsPbI_3_ (QDs)	n_e_ = 0.64×10^15^	n_e_ = 0.5×10^15^	Choline ligands and 2-pentanol solvent	^131^
SCLC	β-CsPbI_3_ (QDs)	0.62×10^15^	0.34×10^15^	(CH_3_)_3_SI Lewis acid passivation	^132^
SCLC	γ to β-CsPbI_3_	2.09×10^16^	1.84×10^16^	PEA_2_PbI_4_ substrate	^149^
SCLC	γ-CsPbI_3_	7.89×10^16^	5.41×10^16^	Formamidine acetate	^150^
SCLC	β-CsPbI_3_	n_h_ = 7×10^15^	n_h_ = 4.02×10^15^	Incorporation of 3, 5-difluorobenzoic acid hydrazide	^107^
SCLC	γ-CsPbI_3_	n_e_ = 2.48×10^16^	n_e_ = 1.95×10^16^	Ge doping	^151^
TAS	β-CsPbI_3_	n_h_ = 5.96×10^15^	n_h_ = 3.83×10^15^	1,4-butanediamine surface passivation	^152^
SCLC	β-CsPbI_3_	9.51×10^15^	6.19×10^15^	4-aminothiophenol incoporation	^109^
SCLC	γ-CsPbI_3_	n_h_ = 1.66×10^15^	n_h_ = 7.39×10^14^	Propylamine hydrochloride surface treatment	^153^
SCLC	γ-CsPbI_3_	n_e_ = 4.12×10^15^	n_e_ = 1.4×10^15^	Phenyltrimethylammonium iodide	^126^
TAS	γ-CsPbI_3_	2.07×10^16^	1.79×10^16^	Dimethylamine acetate	^108^
SCLC	γ-CsPbI_3_	n_e_ = 7.44×10^15^	n_e_ = 4.76×10^15^; n_e_ = 2.97×10^15^	2,2-dithienylketone; (1,2-di(thiophen-2-yl)ethane- 1,2-dione	^127^
SCLC	γ-CsPbI_3_	n_e_ = 2.94×10^15^	n_e_ = 0.65×10^15^	[6,6]-phenyl-C61-butyric acid methyl ester (PCBM) and 2-fluoro-1,4-phenylenediammonium iodide interface passivation	^154^

SCLC and TAS are abbreviations for space-charge-limited current and thermal admittance spectroscopy, respectively.

However, high trap densities have also been reported for composite films. For example, CsPb_1-x_Sn_x_I_3_ contained a high trap density due to the presence of a high density of Sn vacancies^41^. In addition to composition alloying, longer charge carrier lifetimes and smaller hysteresis were also observed when PEAI was used as a surface capping agent to stabilize the CsPbI_3_ α-phase, which originated from passivation by surface-coordinating PEA^+^ organic cations^66^. A similar defect reduction in CsPbI_3_ was effected with phenyltrimethylammonium chloride (PTACl) passivation^62^, indicating that the benefit of this approach could be extended to other halide perovskites. Additional examples of defect passivation following phase stabilization are provided in Table 1. For example, B/X site doping with smaller ions normally induces intrinsic defect suppression due to stronger binding of the ions. In contrast, organic ligand treatment usually passivates extrinsic surface defects. More specifically, since 2022, much attention has been paid to defect passivation. The black phase of CsPbI_3_ became relatively stable with HPbI_3,_ and the efficiency was enhanced by surface/interface passivation with organic ligands, such as 3,5-difluorobenzoic acid hydrazide^107^, p-trifluoromethyl phenethylamonium iodide^107^, dimethylamine acetate^108^, and 4-aminothiophenol]^109^. Hence, the stability of the black phase CsPbI_3_ was improved after passivation due to the protection provided by the organic layer at the surface^110^. It was observed that stabilizing the black phase passivated defects at the surfaces of the CsPbI_3_ crystals. Moreover, defect passivation concurrently improved the phase stability of black CsPbI_3_. Overall, the organic molecules bonded to the crystal surface or dopant ions inside the lattice simultaneously increased the phase stability and passivated defects via charge compensation through electrostatic interactions and local strain effects. The pristine trap densities and reduced trap densities after passivation are depicted in Table 1. It should be noted that there was a correlation between the type of passivator molecule and the efficiency of defect passivation. In principle, two binding sites at the passivating agent would be more effective, indicating that two types of defects were passivated at the same time. For example, a sulfonic zwitterion (zwitterion 3-aminopropanesulfonic acid) passivated both deep (uncoordinated Pb^2+^, metallic lead) and shallow (uncoordinated FA^+^, vacancy I^–^) defects via electrostatic coordination and hydrogen bond formation^111^. In addition, stronger bonding with uncoordinated ions at the perovskite surface was more effective for trap passivation. For example, within a series of hydrazide derivatives, including formohydrazide and benzamide, benzoyl hydrazine exhibited the best passivation effect due to its strong chemical bonding with Pb^2+^ ions^112^. In addition to passivation of the uncoordinated Pb^2+^ ions, benzoyl hydrazine also formed a hydrogen bond with iodide to assist coordination^112^.

As shown in Table 1, SCLC and TAS are the two most commonly used techniques for determining trap densities. In practice, these two experimental techniques have their own advantages and limitations. The SCLC method is widely used to determine carrier transport properties, including trap concentrations and charge mobilities. However, the trap densities determined in this way are imperfect and underestimated, as this estimation is based on the assumption of a J-V linear dependence in the ohmic region until all defects are filled. An onset might result from ionization defects or a double injection effect. By analyzing the capacitance changes with alternating current (AC) voltage, defect features involving the energy level of the defect and its density can be deduced. However, the limitations of TAS include the following: i) Overestimation of defect densities in cases of shapes and densities similar to those of the valence and conduction band states. ii) Only the traps with energies below the energy demarcation point can be counted, leading to omission of deeper traps. iii) Some trapped charges with long thermal emission times could be ignored, ultimately resulting in inaccurate quantification of the trap density^113^.

## Stabilization management progress and photovoltaic efficiency trend of black phase CsPbI_3_

**Fig. 6:**
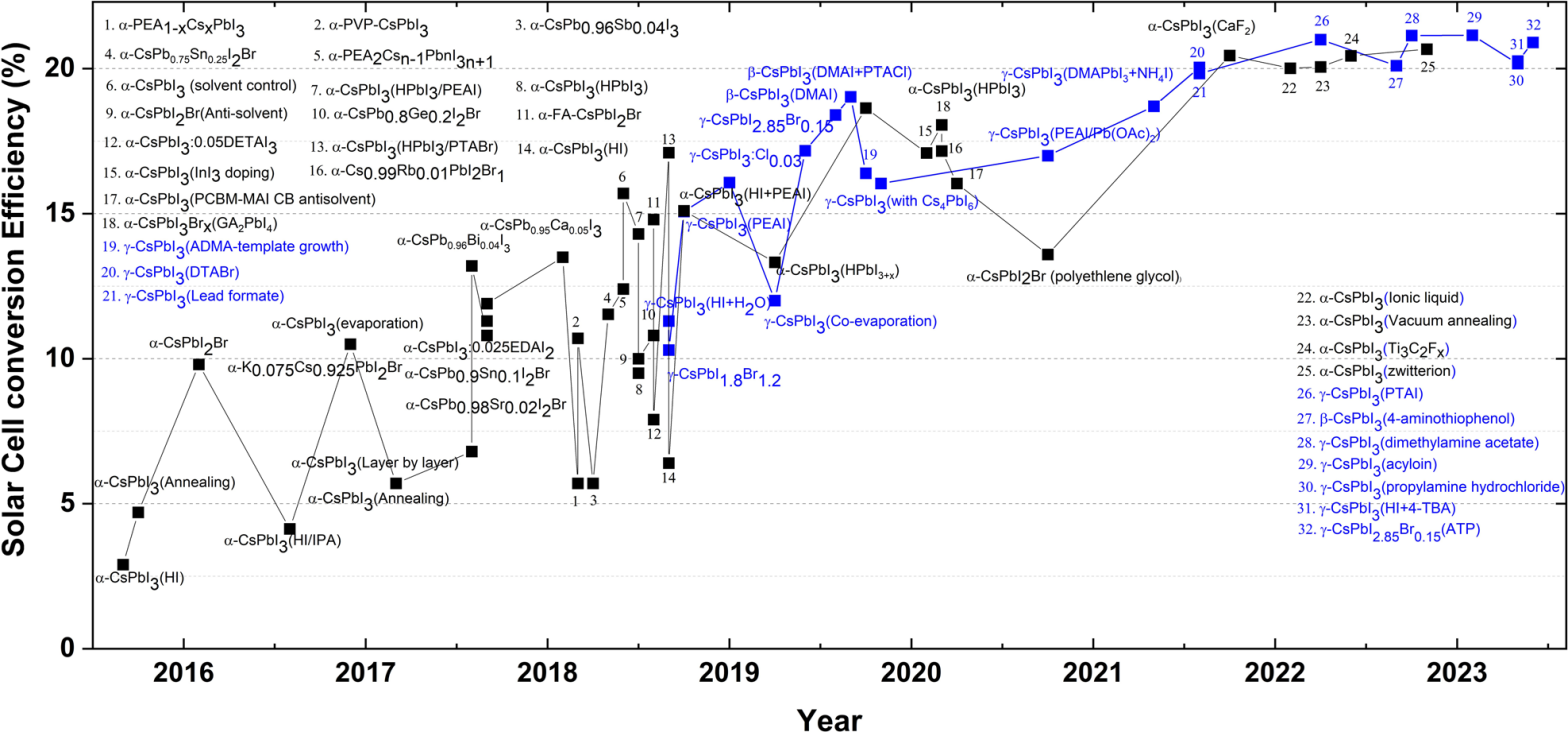
Stabilization management progress and photovoltaic efficiency trend of black phase CsPbI_3_. Overview of recent progress in CsPbI_3_ perovskite solar cell efficiencies in the α^37,39,45,57,66,67,75,94,95,97,110,115,117–121,123,125,143,148,155–169^ (black) and β/γ^23,24,62,75,85,101,105,108,109,114–116,122,124,126,127,134,153,170^ (blue) phases, identifying key technological advances.

**Fig. 7:**
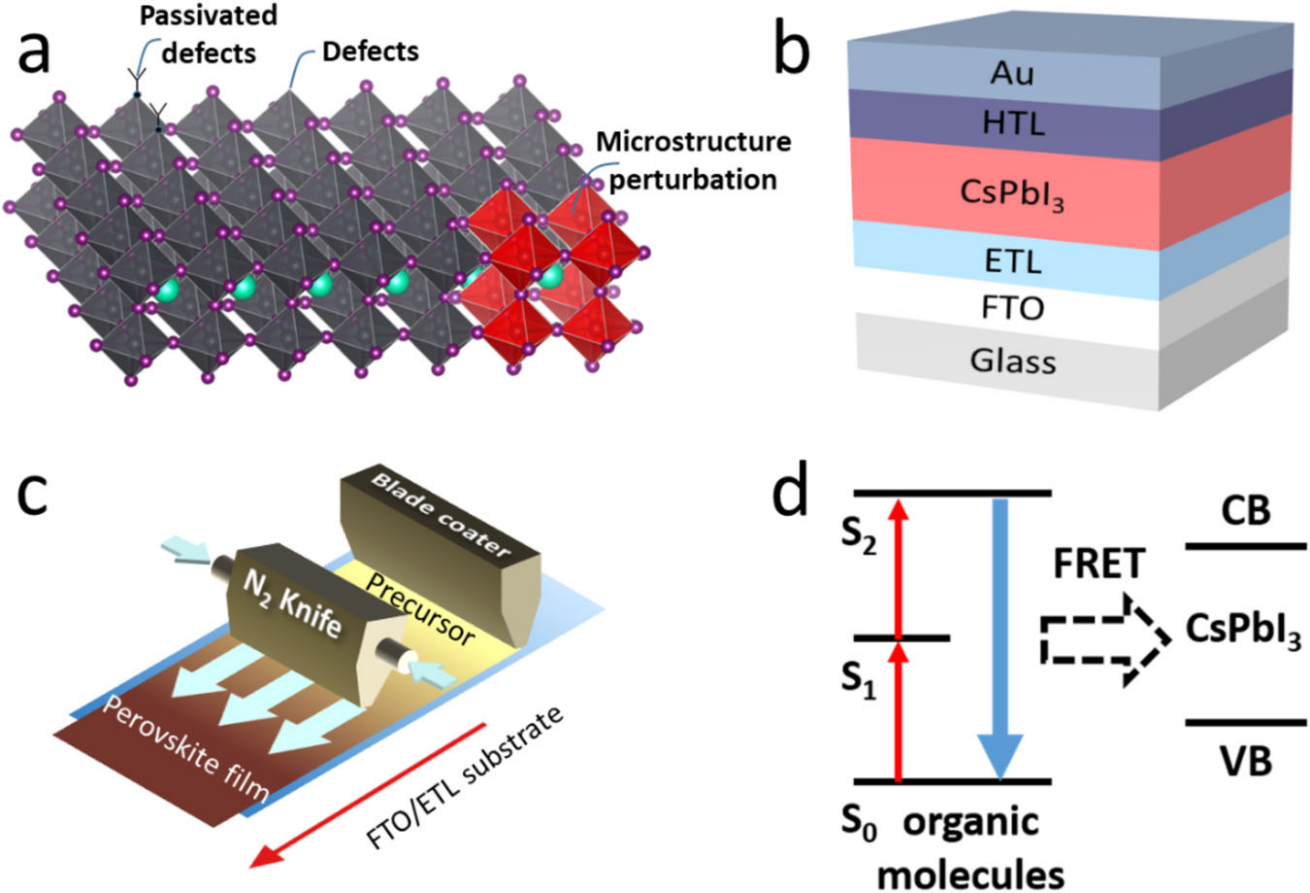
Potential future research directions for CsPbI_3_ solar cells. **a** Diagram of defects, passivated defects, and microstructure perturbation inside/on perovskite crystals. **b** Generic structure of perovskite solar cells. **c** Schematic of the blade coating technique. **d** Förster resonance energy transfer (FRET) between organic molecules and perovskite materials. The red and blue arrows represent low-energy (long wavelength) and high-energy (short wavelength) photons, respectively. S_0_, S_1_, and S_2_ refer to the ground state, lowest excited state, and a higher excited state, respectively.

The PCEs of CsPbI_3_-based solar cells have swiftly increased to more than 20% (Fig. 6) since Eperon et al. reported the first CsPbI_3_ PSC with a 2.9% PCE in 2015^56^. However, phase stability remains a critical issue for commercial electronic devices. After the first cubic phase CsPbI_3_ solar cells were fabricated in 2015^56^, there appeared to be two trends for increasing the conversion efficiencies of CsPbI_3_-based solar cells. Between 2015 and 2018, considerable effort, such as doping, nanocrystal syntheses, and the use of additives and surface functionalization, was expended to obtain stable cubic-phase CsPbI_3_, which led to massive gains in device performance. However, since 2018, the research focus has shifted to stabilizing the CsPbI_3_ β- or γ-phase, as shown in Fig. 7. In 2018, Zhao et al. fabricated high-quality γ-phase CsPbI_3_ thin films by introducing a small amount of H_2_O and tailoring the grain sizes, with an achieved an efficiency of 11.3%^23^. By doping with 40% Br, black γ-phase CsPbI_3_ was obtained with a PSC efficiency of 10.3%^114^. Later, by tuning the amount of PEAI added, the CsPbI_3_ γ-phase exhibited a 15.07% efficiency while maintaining the bandgap^115^. Afterward, a 16.07% efficiency was obtained with incorporation of small amounts of Cl ions beginning in 2019^85^. In addition to the relatively lower efficiency (12%) for coevaporation^116^, the record was increased to 17.17% with bromide-doped materials in June 2019^105^. Afterward, nondoping methods (via DMAI additives) to stabilize the β phase afforded an efficiency of 18.4% in August 2019^24^. To date, the best efficiency of 19.03% was recently reported by applying both DMAI additives and PTACl functionalization^62^. Almost simultaneously, an efficiency of 18.64% was reported when HPbI_3_ was used to stabilize α-phase CsPbI_3_^117^. Compared to those of α-phase CsPbI_3_, β/γ-phase CsPbI_3_ solar cells are still in their infancy. Nevertheless, β/γ-phase CsPbI_3_ materials exhibited high efficiencies and promise for future development. Although some effort was made to stabilize the CsPbI_3_ black phase in 2020 and 2021, the highest efficiencies for α- and β/γ-phase CsPbI_3_ solar cells were still obtained in 2019. For example, tuning the tolerance factor via doping, reducing the surface energy by growing smaller crystallites of the inorganic LHP, or incorporating additives, have been explored. In February 2020, a 17.09% efficiency was achieved with the incorporation of InI_3_ into CsPbI_3_^118^. A little later, a 17.16% efficiency was obtained by doping Rb into CsPbI_2_Br^119^. Afterward, a new mediator-antisolvent strategy combining phenyl-C61-butyric acid methyl ester (PCBM) in combination with the chlorobenzene antisolvent and MAI mediator was developed to improve cubic phase stability, and a 16.04% efficiency was realized in April^120^. In October, a lower efficiency of 13.59% was reported when polyethylene glycol was used as an additive^121^. A similar trend was obtained in stabilizing the CsPbI_3_ β/γ-phase. Compared with the highest efficiency reported in 2019, a lower efficiency of 17% was reported for the stable γ-phase obtained by introducing PEA^+^ into the CsPbI_3_ precursor in August 2020^122^. No efficiency records were published in 2020, possibly because phase stability was no longer the main issue limiting the efficiencies of CsPbI_3_ perovskite solar cells. As with the development of organic‒inorganic LHP solar cells, the focus shifted toward trap management. For this purpose, reducing the trap population and the negative impacts of traps on devices constitutes a future pathway for increasing the efficiencies of CsPbI_3_ perovskite solar cells. Although the trap density was partly suppressed when stabilizing the black phase, the extent of defect passivation was still not sufficient for better performance. Figure 6 shows that in 2021, attention shifted toward passivating the defects in α-phase CsPbI_3-x_Br_x_ solar cells^123^. By using a spontaneous interfacial manipulation method to passivate the interface and surface defects with the formation of a 2D guanidinium lead iodide (GA_2_PbI_4_) layer on top of the 3D perovskite layer, a significant improvement in efficiency from 13.64% to more than 18% was obtained with the elimination of interfacial defects^123^. Moreover, 81% of the initial efficiency was retained after the solution was kept in ambient air for more than 1000 hours^123^. In 2021, the highest efficiency for α-CsPbI_3_ PSCs was 20.45%. Similarly, a 20.04% efficiency was reported for β-CsPbI_3-x_Br_x_ after treatment with N,N,N-trimethyl-1-dodecanaminium bromide (DTABr)^124^.

In particular, from 2022 to the present, many researchers have begun to shift their attention to defect passivation of CsPbI_3_ films because the α and β/γ phases have already been stabilized with doping, surface treatments or incorporation of additives. For example, after the incorporation of Ti_3_C_2_F_x_ QDs^125^ or the use of zwitterion salt interface passivation^110^, efficiencies of 20.44% and 20.67%, respectively, were reported for α-CsPbI_3_ thin films. In addition, more than 90% of the initial efficiency was retained by both passivated devices after a period of one month^110,125^. Simultaneously, high-efficiency records were reported for β/γ-phase CsPbI_3_ films after reducing the defect densities. In April 2022, a record efficiency of 21% was reported for γ-phase CsPbI_3_ by Tan et al., who used phenyltrimethylammonium iodide (PTAI) to passivate the surface defects^126^. Later, a higher efficiency of 21.14% was achieved by incorporating dimethylamine acetate (DMAAc) to reduce the defect density^108^. Finally, in February 2023, a record efficiency of 21.15% was reported after the incorporation of an acyloin ligand (1,2-di(thiophen-2-yl)ethane-1,2-dione) into a γ-CsPbI_3_ thin film^127^. Afterward, efficiencies of 20.26% and 20.9% were reported in April and May, respectively, after the introduction of 4-thioureidobenzoic acid (4-TBA) (into γ-CsPbI_3_)^128^ and carboxyethylisothiuronium chloride (ATP) (into γ-CsPbI_2.85_Br_0.15_)^129^. In recent years, work has also been done on improving the conversion efficiencies of CsPbI_3_ QDs/nanocrystals with surface passivators, including guanidinium^130^ and 2-pentanol^131^. However, their efficiencies (~ 16%)^130–132^ were far from the record (~ 21%) for CsPbI_3_ thin films, possibly due to the inevitably high number of grain boundaries. Thus, the efficiencies of CsPbI_3_ QDs/nanocrystals from 2022 onward are selectively excluded from Fig. 6 because these records are not representative of the conversion efficiency trend for CsPbI_3_-based solar cells.

Recently, a large homogeneous dataset of maximum conversion efficiencies was collected and analyzed by tracking operational aging data over the past three years, and it revealed that more efficient devices exhibited greater stabilities than less efficient devices^133^. Two possible explanations were deduced: i) the remaining excess charge in the device could trigger degradation when the efficiency was lowered by transport limitations or (ii) the presence of pinholes and defects decreased the efficiency of the device and simultaneously resulted in low stability^133^. This indicated that improvements in efficiencies and stabilities were not mutually exclusive. In contrast, passivating the defects and seeking more efficient devices are still research foci. This theory paves the way to more efficient CsPbI_3_ solar cells with high stabilities in the future.

## Summary and outlook

In summary, the use of inorganic LHPs had eliminated the chemical instability issues observed with organic‒inorganic LHPs by generating high thermodynamic stabilities toward decomposition to binary halide products. However, structural instability resulting from the relatively low tolerance factor (*t*) is the major bottleneck for developing efficient optoelectronic materials, such as solar cells. We critically reviewed the reported stabilization protocols, which provided feasible solutions for phase stabilization in black phase CsPbI_3_ and lead-free CsSnI_3_. We also discussed the correlations between phase stabilization and defect passivation in these protocols, as well as the growing trend in the efficiencies of black CsPbI_3_-based solar cells. The results revealed that the β/γ phases show more promise for improving the stabilities of inorganic LHP materials and their PV efficiencies, possibly due to their nearly room temperature syntheses. Even though remarkable improvements in phase stability have been achieved, there are still issues to overcome for future commercialization of CsPbI_3_. Here, we envision that the following research directions are very important for improving the stabilities of inorganic LHP materials and their applications:

(i) Rationally reducing defect densities in inorganic LHP thin films. Although the defect densities of inorganic LHPs can be suppressed during phase stabilization, their densities are still much greater than those of traditional semiconductors. Defect passivation with inorganic LHPs can be guided by the valuable experience in passivating harmful defects in organic‒inorganic LHP materials. Continued effort toward doping/alloying, grain-boundary functionalization, and more effective surface passivation will provide high-quality inorganic LHP layers for high-efficiency solar cells.
(ii) Revealing the exact mechanism for degradation of inorganic LHPs during operation. To date, several achievements have been made in realizing relatively stable inorganic LHP solar cells. These materials retain 90% of the initial PCE after 3000 h of continuous operation^134^. However, an efficient operating time is still insufficient for commercialization. Advanced synchrotron-based characterization methods^135^ with high resolution or an integrated luminescence and electron microscopy (iLEM) system^136^ may reveal in-depth nanoscale behavior that helps us understand the degradation mechanism. In fact, the defects, crystalline structures, and microstructural changes that occur during the inorganic LHP degradation process are not fully understood.
(iii) Optimizing the architectures of inorganic LHP solar cells. After addressing the problem of phase stability, the next step is to fabricate new electron-transport-layer/inorganic LHP/hole-transport-layer interfaces and electrode materials. Optimized interface and electrode materials could passivate harmful surface/interface defects and allow better charge extraction. Currently, although the highest efficiency of a CsPbI_3_ solar cell with an ITO/TiO_2_/LHP/Spiro-OMeTAD/Au structure has exceeded 21%^110,127^, defect passivation and band alignment at the hole/electron transport-perovskite interface are still necessary. For example, deposition of a layer of zwitterionic ions (cesium (2 S,3 S)-3-amino-2-methyl-4-oxoazetidine-1-sulfonate) between the electron transport and perovskite layers controlled interface densities and adjusted the band alignment for efficient electron extraction^110^. To date, less attention has been given to this aspect of inorganic LHP-based devices compared with their organic‒inorganic counterparts. Therefore, more stable inorganic LHP solar cells with higher efficiencies could be realized by putting more effort into understanding the contact interface and developing more efficient transport layer materials.
(iv) Innovative techniques for fabricating large-area CsPbI_3_ perovskite films are highly necessary. From a commercial point of view, large-scale fabrication will be the greatest challenge for CsPbI_3_ solar cells. To date, there have been no reports on high-quality large-area CsPbI_3_ perovskite solar cells because of inhomogeneous crystallization of all-inorganic perovskite covering large areas. Therefore, more precisely controlled deposition techniques, such as chemical vapor deposition^137^ or blade coating^138^, should be further developed for large-scale fabrication. For example, blade coating is currently the common technique used in fabricating large organic‒inorganic LHP films^138^. Similarly, blade coating could also be used for preparing large CsPbI_3_ solar cells^139^.

Notably, several strategies can be adopted to improve the conversion efficiencies of CsPbI_3_ solar cells by moving their absorption bands to longer wavelengths. For example, the introduction of specific organic molecules (such as rubrene) in MAPbI_3_ extended the spectral absorption band from the visible to the near-infrared range via triplet-triplet annihilation and Förster resonance energy transfer (FRET)^140^. This strategy could also be applied to CsPbI_3_-based solar cells. Although it is tedious to fabricate CsPbI_3_ solar cells with high efficiencies and long-term phase stabilities, combining methods such as joint additives and surface functionalization has been successful in achieving stable black phase CsPbI_3_. This trend is very similar to that observed in the development of organic‒inorganic perovskite-based solar cells. A selection of strategies developed for stable organic‒inorganic perovskites could guide the generation of the highly stable room-temperature black phase CsPbI_3_. This has ultimately shaped the progress in solar cell efficiencies and will likely continue to play a role in the future. Significantly, through ongoing efforts to stabilize black phase CsPbI_3_ thin films, the PCEs of photovoltaic devices will increase and continue to approach the thermodynamic limit.
